# Meteorological Observations for May, 1857, at Atlanta, Ga.

**Published:** 1857-06

**Authors:** J. G. Westmoreland


### METEOROLOGICAL OBSERVATIONS FOR MAY, 1857, AT ATLANTA, GA.

| MAY. | THERMOMETER. | | | BAROMETER. | | | WIND. | REMARKS. |
|---|---|---|---|---|---|---|---|---|
| | 7 A. M. | 2 P. M. | 7 P. M. | 7 A. M. | 2 P. M. | 7 P. M. | | |
| 1 | 56 | 68 | 64 | 29.90 | 29.90 | 29.90 | E. | Cloudy—Rain ⅛ in. |
| 2 | 66 | 72 | 68 | 29.90 | 29.90 | 29.90 | S. W. | Hazy. |
| 3 | 60 | 62 | 60 | 29.85 | 29.80 | 29 70 | S. W. | Cloudy—Rain 2. |
| 4 | 48 | 68 | 62 | 29.80 | 29.75 | 29.75 | S. W. | Cloudy. |
| 5 | 48 | 72 | 62 | 29.80 | 30. | 29.95 | W. | Fair. |
| 6 | 56 | 54 | 52 | 29.90 | 29.90 | 29.85 | N. | Cloudy—Rain ¼. |
| 7 | 50 | 68 | 60 | 29.90 | 29.95 | 30. | S. W. | Fair. |
| 8 | 52 | 75 | 60 | 30.08 | 30.18 | 30.18 | W. | Fair. |
| 9 | 68 | 77 | 65 | 30.20 | 30.24 | 30.15 | S. W. | Fair. |
| 10 | 58 | 86 | 78 | 30. | 30. | 29.95 | W. | Fair. |
| 11 | 55 | 75 | 64 | 29.95 | 30. | 29.90 | S. W. | Hazy. |
| 12 | 56 | 78 | 74 | 29 95 | 30.05 | 30.02 | S. W. | Fair. |
| 13 | 60 | 66 | 64 | 30.05 | 30. | 30. | S. E. | Cloudy—Drizzly. |
| 14 | 62 | 78 | 70 | 29.90 | 29.90 | 29.85 | S. E. | Hazy. |
| 15 | 60 | 78 | 68 | 29.70 | 29.83 | 29.85 | W. | Cloudy—Rain ¼. |
| 16 | 56 | 64 | 60 | 29.83 | 29.85 | 29.90 | N. W. | Hazy. |
| 17 | 60 | 64 | 60 | 29.90 | 29.85 | 29.90 | S. | Cloudy—Rain ½. |
| 18 | 50 | 56 | 52 | 29.65 | 29.65 | 29.65 | S. E. | Cloudy—Rain 6-8. |
| 19 | 48 | 56 | 48 | 29.70 | 29.80 | 29.80 | N. W. | Cloudy. |
| 20 | 38 | 50 | 52 | 29.90 | 29.95 | 29.95 | N. W. | Fair. |
| 21 | 40 | 62 | 54 | 30. | 30. | 30. | N. W. | Fair. |
| 22 | 40 | 70 | 60 | 30.05 | 30.10 | 30.05 | W. | Fair. |
| 23 | 50 | 78 | 64 | 30.05 | 30.15 | 30.10 | W. | Fair. |
| 24 | 60 | 82 | 64 | 30.05 | 30.15 | 30.05 | S. W. | Fair. |
| 25 | 64 | 80 | 72 | 30.05 | 30.05 | 30. | S. W. | Fair. |
| 26 | 60 | 70 | 68 | 29.95 | 30. | 30. | W. | Hazy. |
| 27 | 60 | 76 | 68 | 29.95 | 30. | 29.95 | S. W. | Cloudy—Rain 1-16. |
| 28 | 54 | 78 | 60 | 29.95 | 29.95 | 29.95 | W. | Fair. |
| 29 | 62 | 78 | 70 | 30. | 29.95 | 29.95 | W. | Fair. |
| 30 | 64 | 84 | 78 | 30. | 29.95 | 29.95 | W. | Fair. |
| 31 | 70 | 80 | 68 | 30. | 30. | 29.90 | W. | Hazy—Drizzly. |

Furnished by

J. G. WESTMORELAND, M. D.

